# A Comparative Study of the Effectiveness and Safety of Topical Calcipotriol and Topical Methotrexate in Chronic Plaque Psoriasis

**DOI:** 10.7759/cureus.59878

**Published:** 2024-05-08

**Authors:** Kamal Das, Rajesh Ranjan, Prabhat Kumar, Satish Chandra

**Affiliations:** 1 Pharmacology, Rajendra Institute of Medical Sciences, Ranchi, IND; 2 Dermatology, Rajendra Institute of Medical Sciences, Ranchi, IND

**Keywords:** vitamin d, pasi score, methotrexate, calcipotriol, psoriasis

## Abstract

Background

Psoriasis is a papulosquamous disease with variable morphology, distribution, severity, and course. Chronic plaque psoriasis, or psoriasis vulgaris, is the most common form of psoriasis. Present available preparations for mild to moderate chronic plaque psoriasis for topical use are local corticosteroids, coal tar, dithranol, tazarotene, calcipotriol, tapinarof, and calcineurin inhibitors. However, every preparation has its disadvantages. Calcipotriol, an active form of vitamin D, is available in topical form for dermatological use. Chronic plaque psoriasis is the chief medical use of calcipotriol for mild to moderate form. Methotrexate has dramatic results in psoriasis when used systemically. Now, topical formulation is being advocated in localized psoriasis, which is not associated with the side effects of the systemic form. Therefore, this study aimed to compare the effectiveness of topical calcipotriol and topical methotrexate on the basis of the psoriasis area severity index (PASI) in patients of chronic plaque psoriasis and compare their safety in terms of adverse effects.

Methodology

The total number of patients included in the study was 60. They were divided into two groups, with 30 patients each. One group was prescribed ointment calcipotriol 0.005% twice daily local application (Group C). The other group was prescribed methotrexate gel 1% twice daily local application (Group M). The patients were followed up on the fourth and eighth weeks, and at each time, thorough clinical examinations were conducted for all patients. The PASI score was calculated in each patient every time. Safety was assessed by biochemical parameters, and tolerability was assessed by the incidence of adverse effects. All the patients included in the study were investigated at baseline, fourth week, and eighth week. The data collected were transferred to a master chart and analyzed.

Results

For the patients in group C, the mean PASI score at 0 week was 5.93 ± 2.62, while at four weeks, the mean PASI score declined to 1.67 ± 1.13, and at eight weeks, the mean PASI score further declined to 0.67 ± 0.68. For the patients in group M, the mean PASI score at 0 week was 5.91 ± 2.22, while at four weeks, the mean PASI score declined to 1.91 ± 1.11, and at eight weeks, the mean PASI score further declined to 0.89 ± 0.72. Furthermore, there was no significant difference in the mean PASI score at various time points when compared between the two groups (p-value = 0.761, 0.296, 0.079, respectively). Thus, both drugs seem to be effective in treating mild- to moderate-grade chronic plaque psoriasis. Most of the patients in both groups showed marked clearance of the lesions. However, there were six patients in the calcipotriol group showing complete clearance of the lesions having mild-degree plaque psoriasis, as compared to three patients in the methotrexate group. In the present study, based on the comparison of safety and tolerability, four out of 30 patients (13.3%) in the calcipotriol group suffered skin irritation, whereas six out of 30 patients (20%) in the methotrexate group complained of a burning sensation. The adverse effects seen in the patients were transient and mild.

Conclusion

Topical calcipotriol and methotrexate were effective in reducing lesions in patients with chronic mild to moderate plaque psoriasis. Both drugs were well tolerated with mild and transient adverse effects and did not alter hematological and biochemical parameters.

## Introduction

Psoriasis is a papulosquamous disease with variable morphology, distribution, severity, and course. Its presence is at typical sites of well-defined, erythematous, indurated papules and plaques, which are surrounded by large, loose, silvery scales [1]. The worldwide prevalence is about 2%. The prevalence of psoriasis in India ranges from 0.44% to 2.8%. Although patients can develop psoriasis at any age, the highest incidence was noted in the age group of 20-39 years. It is twice more common in males compared to females. Most patients are in their third or fourth decade at the time of presentation [2]. Psoriasis is classified based on its onset, evolution, and morphology into chronic plaque psoriasis, guttate psoriasis, pustular psoriasis, inverse psoriasis, and erythrodermic psoriasis. Chronic plaque psoriasis, also called psoriasis vulgaris, is the most common form of psoriasis. The classical clinical manifestations are sharply demarcated, erythematous, pruritic plaques covered in silvery scales. The lesions are bilateral and symmetrical over pressure points (elbows and knees), extensors, scalp, and trunk. Palms and soles are also frequently involved.

Present available preparations for mild to moderate chronic plaque psoriasis for topical use are local corticosteroids, coal tar, dithranol, tazarotene, calcipotriol, tapinarof, and calcineurin inhibitors like tacrolimus and pimecrolimus. However, every preparation has its disadvantages. Topical steroids can cause dermal atrophy, tachyphylaxis, perilesional hypopigmentation, and early relapse. Coal tar is cruder, blacker, and messy to use. It can also cause chemical folliculitis and irritant contact dermatitis and works in association with light therapy. Dithranol is an irritant and stains clothes and surrounding skin, and it also works with light therapy. Tazarotene is not routinely used in chronic plaque psoriasis; it is an irritant and contraindicated in pregnancy [3]. Tapinarof is recently approved and is not available in India. Tacrolimus and pimecrolimus are reserved for facial lesions.

Calcipotriol, also known as calcipotriene, is a synthetic derivative of calcitriol, an active form of vitamin D. It is available in topical form for dermatological use. Chronic plaque psoriasis is the chief medical use of calcipotriol for mild to moderate form [4]. Calcipotriol inhibits epidermal cell proliferation and enhances cell differentiation. Calcipotriol is a first-line drug for mild to moderate psoriasis alone or with another anti-psoriatic drug. It is applied twice daily, which is efficacious and safe for the treatment of psoriasis. Clinical improvement of psoriatic lesions occurs within two weeks, with maximal benefits observed in four to eight weeks. It is used as 0.005% (50 mcg/gram) ointment/cream [5]. Studies have shown that calcipotriol has an excellent safety profile. Adverse reactions are mild, which are irritation of the skin (lesional and perilesional), pruritus, burning sensation, erythema, and scaling. Calcipotriol should not be used on the face [6].

Methotrexate is a folic acid antagonist with cytotoxic and immunosuppressant activity. It is being used in different conditions like cancer chemotherapy, rheumatoid arthritis, Crohn’s disease, and ectopic pregnancy. Methotrexate is usually given orally. Now, topical formulation is available for dermatological use [7]. It is actively taken up into cells by the folate transport system and is metabolized to polyglutamate derivatives. These polyglutamate derivatives are retained in the cells for weeks and months even in the absence of an extracellular drug [8]. Methotrexate has dramatic results in psoriasis when used systemically. Topical formulation is being advocated in localized psoriasis, which is not associated with the side effects of the systemic form. Topical methotrexate acts through a local inhibition of DNA synthesis in the psoriatic hyperplastic epidermis [9]. Clinical improvements were observed in maximum patients when used for eight weeks with twice-daily application [10]. The most common adverse reactions for topical methotrexate are burning sensation, irritation, pruritus, and redness.

To date, there are very few studies that have evaluated the effects of topical calcipotriol and topical methotrexate. There is no study comparing both drugs in the same studies. Hence, this study was designed to compare the effectiveness and safety of topical calcipotriol and topical methotrexate in chronic plaque psoriasis.

## Materials and methods

This study was conducted in the Department of Pharmacology and Therapeutics in collaboration with the Department of Skin, STD, and Leprosy at Rajendra Institute of Medical Sciences (RIMS), Ranchi, India, among patients with chronic plaque psoriasis attending the skin OPD during the study period. It was an observational, randomized, open-label, parallel, effectiveness, and safety study. A simple randomization method was adopted to randomize the patients into two groups using Microsoft Office Excel (Microsoft Corporation, USA). The Institutional Ethics Committee of RIMS, Ranchi, issued approval (ref. no. 182, dated December 21, 2019).

Sample size

Sixty (60) patients who had mild to moderate (psoriasis area severity index (PASI) up to 15) chronic plaque psoriasis and met the inclusion criteria with no reason for exclusion were included in the study.

Inclusion and exclusion criteria

The inclusion criteria include patients who had been diagnosed with chronic plaque psoriasis, patients with psoriatic lesions less than 25% of the body surface area (PASI up to 15), those who were not taking oral treatment for psoriasis or receiving injections as treatment for psoriasis, 18 years of age or older, and male and female.

The exclusion criteria are patients with psoriatic lesions on the face and/or scalp; administration of topical, systemic, or intrathecal therapy or UV radiation for at least two months prior to the study; pregnant or lactating mother; patients with evidence of hepatic and/or renal impairment; and history of hypersensitivity to any study drug.

Study duration

For the individual patient, the scheduled treatment duration was eight weeks following selection, and the total duration of the study was 20 months after getting clearance from the ethical committee. The study was conducted from December 24, 2019, to August 23, 2021.

Procedure

The patients were divided into two random groups. One group was prescribed ointment calcipotriol 0.005% with twice-daily local application. The other group was prescribed methotrexate gel 1% twice-daily local application. The patients were followed up on the fourth and eighth weeks, and at each time, thorough clinical examinations were conducted for all patients. The PASI score was calculated for each patient every time.

The formula for the PASI score is as follows: PASI = 0.1(Eh+Ih+Dh) Ah + 0.2(Eu+Iu+Du) Au + 0.3(Et+It+Dt) At + 0.4(El+Il+Dl) Al, where E stands for erythema, I for induration, D for desquamation, h for head, u for upper limb, t for trunk, l for lower limb, and A for area score.

Safety was assessed by biochemical parameters, and tolerability was assessed by the incidence of adverse effects, which was compared with the standard side effect checklist prepared for both drugs (Appendix A). All the patients included in the study were investigated at baseline, fourth week, and eighth week. Investigations included complete blood count, liver function test (total bilirubin, serum glutamic oxaloacetic transaminase (SGOT), and serum glutamate pyruvate transaminase (SGPT)), renal function test (serum urea and creatinine), and serum calcium. 

Statistical analysis

The data collected were transferred to a master chart. Statistical testing was conducted using the SPSS Statistics for Windows, version 17.0 (released 2008, SPSS Inc., Chicago). Continuous variables were presented as mean ± standard deviation (SD) or median (interquartile range (IQR)) for non-normally distributed data. Categorical variables were expressed as frequencies and percentages. The comparison of normally distributed continuous variables between the groups was performed using Student’s t-test. Nominal categorical data between the groups were compared using the Chi-squared test or Fisher’s exact test as appropriate. Non-normally distributed continuous variables were compared using the Mann-Whitney U test. For all statistical tests, a p-value less than 0.05 was taken to indicate a significant difference.

## Results

This study was conducted from December 24, 2019, to August 23, 2021. The number of patients meeting the inclusion and exclusion criteria was 65 (male = 42, female = 23). The number of patients lost to follow-up was 5. The number of patients who completed the study was 60. The number of patients in Group C (calcipotriol group) was 30 (male = 20, female =10). The number of patients in group M (methotrexate group) was 30 (male =20, female =10).

Table 1 depicts the comparison of the mean age of the patients between the two groups under the study. It was observed that the mean age of the patients in group C was 30.13 ± 7.470 years, while the mean age of the patients in group M was 29.67 ± 8.03 years. Furthermore, there was no significant difference in the mean age of the patients when compared between the two groups (p-value = 0.817).

**Table 1:** Comparison of mean age of the patients between the two groups Test used: Student's t-test

	Group C (n = 30)	Group M (n = 30)	p-value
	Mean ± SD	Mean ± SD	
Age (years)	30.13 ± 7.470	29.67 ± 8.03	0.817

Table 2 depicts the comparison of the age distribution of the patients between the two groups under the study. It was observed that under the group C, 46.7% of the patients were in the age group of 21-30 years, 36.7% were in the age group of 31-40 years, 10% were in the age group of 19-20 years, and 6.7% were in the age group of 41-50 years. Under group M, 50% of the patients were in the age group of 21-30 years, 26.7% were in the age group of 31-40 years, 13.3% were in the age group of 19-20 years, and 10% were in the age group of 41-50 years. Furthermore, there was no significant difference in the age distribution of the patients when compared between the two groups (p-value = 0.837). 

**Table 2:** Comparison of age groups distribution between the two groups Test used: Chi-square test

Age groups	Group C		Group M		p-value
	Frequency	%	Frequency	%	
19-20 years	3	10.0%	4	13.3%	0.837
21-30 years	14	46.7%	15	50.0%	
31-40 years	11	36.7%	8	26.7%	
41-50 years	2	6.7%	3	10.0%	
Total	30	100%	30	100%	

Table 3 depicts the comparison of the sex distribution of the patients between the two groups under the study. Under both groups, 33.3% of the patients were females and 66.7% were males. Furthermore, there was no significant difference in the sex distribution of the patients when compared between the two groups (p-value = 1.000). 

**Table 3:** Comparison of the sex distribution between the two groups Test used: chi-square test

Sex	Group C		Group M		p-value
	Frequency	%	Frequency	%	
Female	10	33.3%	10	33.3%	1.000
Male	20	66.7%	20	66.7%	
Total	30	100%	30	100%	

Table 4 depicts the comparison between the two groups under the study according to the mean PASI score at various time points. For the patients in group C, the mean PASI score at 0 week was 5.93 ± 2.62, while at four weeks, the mean PASI score declined to 1.67 ± 1.13, and at eight weeks, the mean PASI score further declined to 0.67 ± 0.68. For the patients in group M, the mean PASI score at 0 week was 5.91 ± 2.22, while at four weeks, the mean PASI score declined to 1.91 ± 1.11, and at eight weeks, the mean PASI score further declined to 0.89 ± 0.72. Furthermore, there was no significant difference in the mean PASI score at various time points when compared between the two groups (p-value = 0.761, 0.296, 0.079, respectively). 

**Table 4:** Comparison of the mean PASI score at various time points between the two groups and p-values of the calcipotriol and methotrexate groups at various time intervals Test used: Mann-Witney U test for the comparison between the two groups and Wilcoxon signed-rank test for the within-group analysis for paired comparisons. PASI: psoriasis area severity index

PASI score	Calcipotriol 0.005% ointment			Methotrexate 1% gel			p-value
	Mean ± SD	Median (IQR)	Min-Max	Mean ± SD	Median (IQR)	Min-Max	
Week 0	5.927 ± 2.6240	5.4 (3.9-7.28)	1.5-10.8	5.907 ± 2.212	6.30 (4.35-8.03)	1.8-9.0	0.761
Week 4	1.670 ± 1.128	1.5 (0.88-2.40)	0-4.5	1.913 ± 1.111	1.80 (1.00-2.65)	0.0-4.5	0.296
Week 8	0.673 ± 0.683	0.55 (0.20-0.65)	0-2.4	0.890 ± 0.719	0.60 (0.48-1.20)	0.0-2.8	0.079
p values							
Week 0-week 4	<0.001**			<0.001**			
Week 0-week 8	<0.001**			<0.001**			
Week 4-week 8	<0.001**			<0.001**			
**signifies highly significant p-value < 0.001							

Table 5 depicts the comparison between the two groups under the study according to the adverse effects on patients at four weeks. In group C, 86.7% of the patients had no adverse effects while 13.3% of the patients had skin irritation. Under group M, 80% of the patients had no adverse effects, while 20% of the patients had burning sensations. Furthermore, there was a significant difference in the distribution of the patients according to adverse effects at four weeks when compared between the two groups (p-value = 0.006). 

**Table 5:** Comparison of adverse effects to patients at four weeks between the two groups * significant p-value <0.05. Test used: Chi-square test

Adverse effects at four weeks	Group C		Group M		p-value
	Frequency	%	Frequency	%	
Nil	26	86.7%	24	80.0%	0.006*
Burning sensation	0	0.0%	6	20.0%	
Skin irritation	4	13.3%	0	0.0%	
Total	30	100%	30	100%	

Table 6 depicts the comparison between the two groups under the study according to the adverse effects on patients at eight weeks. In group C, 86.7% of the patients had no adverse effects, while 13.3% of the patients had skin irritations. In group M, 83.3% of the patients had no adverse effects, while 16.7% of the patients had burning sensations. Furthermore, there was a significant difference in the distribution of the patients according to adverse effects at eight weeks when compared between the two groups (p-value = 0.011). 

**Table 6:** Comparison of adverse effects at eight weeks between the two groups *highly significant p-value <0.05. Test used: Chi-square test

Adverse effects at eight weeks	Group C		Group M		p-value
	Frequency	%	Frequency	%	
Nil	26	86.7%	25	83.3%	0.011*
Burning sensation	0	0.0%	5	16.7%	
Skin irritation	4	13.3%	0	0.0%	
Total	30	100%	30	100%	

Table 7 depicts the comparison between the two groups under the study according to the outcomes of investigations at various time points. For both groups C and M, 100% of the patients had favorable outcomes of investigations, i.e., investigations were within normal limits (WNL) at all time points.

**Table 7:** Comparison of investigation findings between the two groups The following investigations were done at baseline, fourth week, and eighth week: complete blood count, liver function test (total bilirubin, serum glutamic oxaloacetic transaminase (SGOT), serum glutamate pyruvate transaminase (SGPT), renal function test (serum urea and creatinine), and serum calcium. Test used: Chi-square test. WNL: within normal limit

Investigations		Group C		Group M		p-value
		Frequency	%	Frequency	%	
0 Week	WNL*	30	100.0%	30	100.0%	–
4 Week	WNL	30	100.0%	30	100.0%	–
8 Week	WNL	30	100.0%	30	100.0%	–

## Discussion

The study was carried out to observe the effectiveness and safety of 0.005% calcipotriol ointment and 1% methotrexate gel in patients having mild to moderate chronic plaque psoriasis. Both drugs were applied in twice-daily dosing. For better comparison, the patients were randomized. Out of 65 enrolled patients, 60 completed the study, and five were lost to follow-up.

Age and sex distribution

Okhandiar et al. in 1963, Bedi et al. in 1995, and Kaur et al. in 1997 observed that the peak onset of the disease was in the third and fourth decades of life [11,12,13]. In this study, the peak onset of the disease was similar to that in other studies. There was male predominance in both groups: group C had 20 males (66.7%) and group M had 20 males (66.7%) in the present study. Okhandiar et al. in 1963 observed that the overall male:female ratio was 2.46:1 [11]. Kaur et al. in 1997 observed that the overall male:female ratio was 2.03:1 [13]. Bedi et al. in 1995 observed that the overall male:female ratio was 2.4:1 [12]. In this study, male predominance was seen, similar to other studies.

Effectiveness

Both drugs seem to be effective in treating mild- to moderate-grade chronic plaque psoriasis. Most of the patients in both groups showed marked clearance of the lesions. However, there were six patients in the calcipotriol group showing complete clearance of the lesions and having mild-degree plaque psoriasis, as compared to three patients in the methotrexate group. The comparison between calcipotriol 0.005% ointment and methotrexate 1% gel did not show any significant difference in their effects on PASI scores at any time point, i.e., week 0 (p-value = 0.761), week 4 (p-value = 0.296), and week 8 (p-value = 0.079). However, within each treatment group, there was a notable improvement from baseline, indicating the efficacy of both treatments in reducing psoriasis severity. Calcipotriol demonstrated a substantial decrease in PASI scores from a mean of 5.927 at week 0 to 1.670 at week 4 and further to 0.673 at week 8 (p-value < 0.001 for all comparisons). This suggests significant improvement in psoriasis severity over time with calcipotriol treatment (p-value <0.001). Similarly, methotrexate showed a marked decrease in PASI scores from a mean of 5.907 at week 0 to 1.913 at week 4 and to 0.890 at week 8 (p < 0.001 for all comparisons), indicating a significant improvement in psoriasis severity over time with methotrexate treatment (p-value < 0.001).

In summary, although there was no significant difference between the two treatments, both calcipotriol and methotrexate exhibited substantial improvements in PASI scores from baseline at all time points, highlighting their efficacy in managing psoriasis. This may signify the clinically significant outcome of calcipotriol although the results of the outcome of effectiveness were statistically insignificant.

Alex et al. in 2020, Parker et al. in 2021, and El Gayar et al. in 2022 also observed marked improvement in PASI scores among patients with chronic plaque psoriasis after applying 0.005% calcipotriol ointment [14,15,16]. Ozcan et al. in 2020 and Chaiyabutr et al. in 2022 observed global improvement of lesions of chronic plaque psoriasis patients treated with 1% methotrexate gel [17,18]. 

Safety and tolerability

In the present study, based on the comparison of safety and tolerability, four out of the 30 patients (13.3%) in the calcipotriol group suffered skin irritation, whereas six out of the 30 patients (20%) in the methotrexate group complained of burning sensations. The adverse effects seen in the patients were transient and mild in nature. No patient was withdrawn from the study due to adverse effects. When we compared the adverse effects of drugs at fourth and eighth weeks, the results were statistically significant. In a study conducted by Murdoch et al., it was found that around 10-15% of patients receiving topical calcipotriol suffered itching and skin irritations [19]. Murtadha et al. in 2010 found burning sensation, irritation, and pruritus in approximately 10-12% of patients treated with topical methotrexate [17]. In the present study, all the patients were investigated for complete blood count, liver function test (total bilirubin, SGOT, and SGPT), renal function test (serum urea and creatinine), and serum calcium at 0 week, fourth week, and eighth week. There was no report of any significant abnormalities in hematology and biochemical laboratory parameters with the twice-daily application of calcipotriol ointment and methotrexate gel. In both groups C and M, 100% of the patients had favorable outcomes of investigations, i.e., investigations were WNL. Murdoch et al. and Murtadha et al. also did not observe any change in hematological and biochemical parameters in patients treated with topical calcipotriol and topical methotrexate, respectively [17,19].

Limitations of the study

The study could have been done with a larger number of patients; a good sample size gives better results. It can also be done for a longer duration, so that adverse effects can be studied in a better way.

## Conclusions

From the findings of this study, we conclude that topical calcipotriol and topical methotrexate were found to be effective in reducing lesions in patients with chronic mild to moderate plaque psoriasis. Both drugs were well tolerated with mild and transient adverse effects. Both drugs also did not alter hematological and biochemical parameters. By using the topical formulation of methotrexate in mild to moderate chronic plaque psoriasis, we may overcome the systemic side effects of methotrexate.

## Appendices

Appendix A 

**Table 8:** Standard side effect checklist for topical calcipotriol and topical methotrexate

Side effects of topical calcipotriol	Side effects of topical methotrexate
Irritation of the skin (lesional and peri-lesional), pruritus, burning sensation, erythema, scaling	Burning sensation, irritation, pruritus, redness
